# 621. Identifying Quality-Improvement Interventions to Improve Inpatient Intravenous Vancomycin Safety at an Academic Medical Center

**DOI:** 10.1093/ofid/ofab466.819

**Published:** 2021-12-04

**Authors:** Sean Christensen, Russell J Benefield

**Affiliations:** University of Utah Health, Salt Lake City, Utah

## Abstract

**Background:**

The reported incidence of intravenous (IV) vancomycin-associated acute kidney injury (AKI) is highly variable. The primary purpose of this study was to determine the baseline rate of IV vancomycin-associated AKI at the University of Utah Hospital (UUH) and Huntsman Cancer Institute (HCI) with the goal of identifying areas of focus for future quality improvement (QI) initiatives.

**Methods:**

This was a retrospective descriptive study of patients ≥ 18 years old, hospitalized at UUH or HCI, who received at least daily scheduled doses of IV vancomycin for ≥ 72 hours between November 1, 2018 and October 31, 2019. AKI was defined using the serum creatinine (SCr) aspect of the AKIN criteria. Variables assessed for association with AKI included demographic characteristics, hospital and unit where vancomycin was initiated, duration of therapy, administration method, and concomitant nephrotoxic medications. Multivariable logistic regression was used to identify variables independently associated with AKI as potential QI interventions.

**Results:**

One thousand eighty-six patients were included. Baseline patient characteristics are listed in Table 1. Throughout our system, 19.7% of patients experienced an AKI while receiving vancomycin. Univariate comparisons are listed in Table 1. Variables independently associated with AKI on multivariable analysis included total body weight (HR 1.02, 95% CI [1.01-1.03]), concomitant administration of calcineurin inhibitors or vasopressors (HR 1.97, 95% CI [1.18-3.29] and HR 1.68, 85% CI [1.07-2.64] respectively), duration of vancomycin therapy (HR, 1.04, 95% CI [1.02-1.06]), and administration in specific units (see Table 1). Administration of vancomycin by continuous infusion showed a protective effect (HR 0.13, 95% CI [0.02-1.12]) as did baseline SCr and total daily dose of vancomycin (HR 0.76, 95% CI [0.61-0.94] and HR 0.63, 95% CI [0.51-0.78] respectively); the latter two are likely a reflection of the study design. The median hospital length of stay in days was longer in individuals experiencing an AKI (19 vs 10, p < 0.0001).

Table 1. Univariate and Multivariate Associations with Vancomycin-Associated Acute Kidney Injury

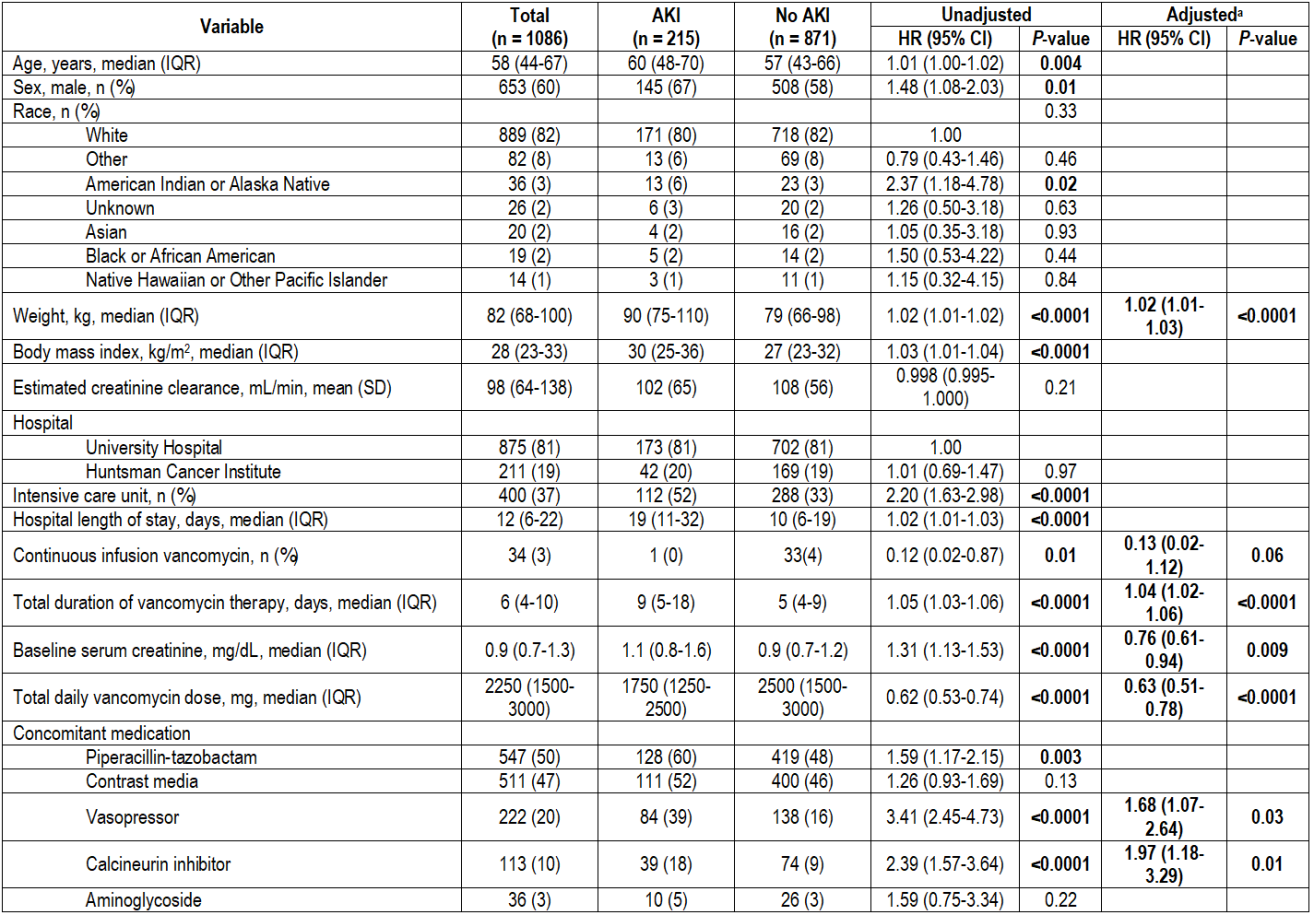

^a^For continuous variables, the HR reported is for each unit increase

Table 1. (Continued) Univariate and Multivariate Associations with Vancomycin-Associated Acute Kidney Injury

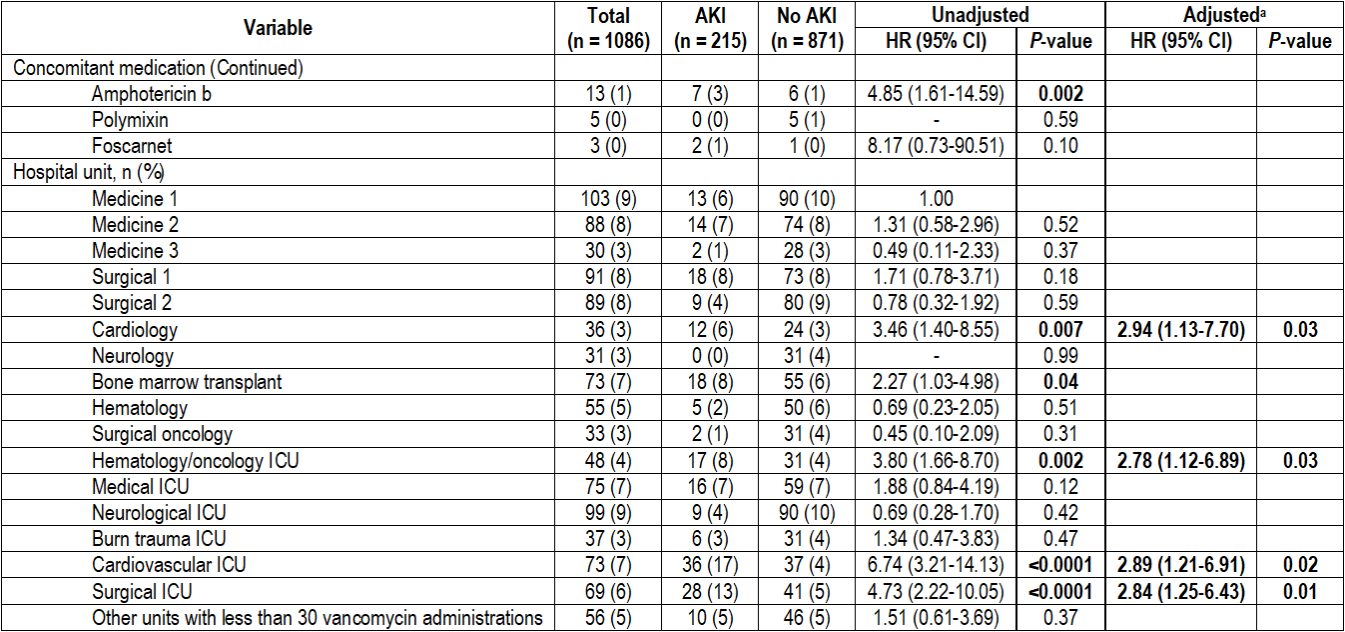

^a^For continuous variables, the HR reported is for each unit increase

**Conclusion:**

Several variables associated with vancomycin-associated AKI within our health system were identified. Future QI interventions to improve vancomycin safety will be pursued.

**Disclosures:**

**Russell J. Benefield, PharmD**, **Paratek Pharmaceuticals** (Grant/Research Support)

